# Complete Chloroplast Genome Features of *Dendrocalamus* *farinosus* and Its Comparison and Evolutionary Analysis with Other Bambusoideae Species

**DOI:** 10.3390/genes13091519

**Published:** 2022-08-24

**Authors:** Jialong Pei, Yong Wang, Juan Zhuo, Huibin Gao, Naresh Vasupalli, Dan Hou, Xinchun Lin

**Affiliations:** 1 State Key Laboratory of Subtropical Silviculture, Zhejiang A&F University, 666 Wusu Street, Hangzhou 311300, China; 2 Yibin Forestry and Bamboo Industry Research Institute, Yibin 644599, China

**Keywords:** *Dendrocalamus farinosus*, Bambusoideae, chloroplast genome, phylogenomics

## Abstract

*Dendrocalamus farinosus* is one of the essential bamboo species mainly used for food and timber in the southwestern region of China. In this study, the complete chloroplast (cp) genome of *D. farinosus* is sequenced, assembled, and the phylogenetic relationship analyzed. The cp genome has a circular and quadripartite structure, has a total length of 139,499 bp and contains 132 genes: 89 protein-coding genes, eight rRNAs and 35 tRNAs. The repeat analyses showed that three types of repeats (palindromic, forward and reverse) are present in the genome. A total of 51 simple sequence repeats are identified in the cp genome. The comparative analysis between different species belonging to *Dendrocalamus* revealed that although the cp genomes are conserved, many differences exist between the genomes. The analysis shows that the non-coding regions were more divergent than the coding regions, and the inverted repeat regions are more conserved than the single-copy regions. Moreover, these results also indicate that *rpoC2* may be used to distinguish between different bamboo species. Phylogenetic analysis results supported that *D. farinosus* was closely related to *D. latiflorus*. Furthermore, these bamboo species’ geographical distribution and rhizome types indicate two evolutionary pathways: one is from the tropics to the alpine zone, and the other is from the tropics to the warm temperate zone. Our study will be helpful in the determination of the cp genome sequences of *D. farinosus*, and provides new molecular data to understand the Bambusoideae evolution.

## 1. Introduction

Bambusoideae is one of the largest subfamilies in the grass family (Poaceae), including 75 genera and 1680 species, which are widely distributed in tropical, subtropical and warm temperate zones [1,2]. Bamboo has a high economic value and is widely used in building materials, in making paper, furniture and musical instruments, and as food, etc. [3]. The traditional bamboo species identification is mainly based on morphological characteristics, such as bamboo shoots and flowers [4]. However, the identification and classification of bamboo has always been a complex problem because many species contain similar morphological characteristics and the flowering interval of most Bambusoideae species is very long. For example, *Phyllostachys edulis* is reported to have a flowering interval of 67 years, *Ph. bambusoides*, 120 years, and *Melocanna baccifera*, 48 years [5]. Therefore, it is difficult to classify Bambusoideae species by morphological characteristics.

*Bambusa*, *Dendrocalamus* and *Gigantochloa* are morphologically similar genera in Bambusoideae. *Bambusa* has more than 100 species, and nearly 80 species are found in southern and southwestern China [1]. *Dendrocalamus* contains 66 species, mainly distributed in tropical and subtropical Asia. Among them, 30 species are found in southwestern China [1,6]. In addition, out of 30 species of Gigantochloa, seven are in China. The classification of bamboo species of *Bambusa*, *Dendrocalamus*, and *Gigantochloa* has long been controversial. In particular, *Gigantochloa* and *Dendrocalamus* can only be distinguished by their filaments [1]. These characteristics make it challenging to differentiate these two genera. In recent years, researchers have continuously provided evidence to prove a difference between them, evidence which is used to classify bamboo species. The flora of China (http://www.iplant.cn/foc/AboutFoc, accessed on 11 January 2022) distinguished the three genera from the size of culm sheath blades and auricles, the length of culm internodes and the branchlets and the morphological characteristics of caryopsis [1]. However, the flowering interval of Bambusoideae is long, and caryopsis acquisition is difficult. Therefore, we need an effective method to assist in the classification of Bambusoideae species. 

The chloroplast (cp) is regarded as the energy source for the evolution of life [7]. Photosynthesis is one of the most important chemical reactions and provides essential energy for most living things on earth [8]. The cp has an independent semi-autonomous extranuclear genome [9]. The cp genome is the crucial genetic material of plants, encoding 100–200 essential genes which encode proteins involved in photosynthesis and other chloroplast functions [10]. Moreover, the cp genomes are ideal tools for studying evolutionary relationships between species because cp genomes are very conserved, and their heredity is independent of the nucleus [11,12]. With the development of high-throughput sequencing technology, every year, numerous plant cp genomes have been sequenced [1]. Therefore, cp genomes have been widely used in plant classification and evolution [9]. Wu et al. (2009) sequenced the cp genomes of *D. latiflorus* and *Bambusa oldhamii*, and believed that cp genomes could be used to provide auxiliary information for the identification and evolution of bamboo species [13]. Many researchers have tried to use the cp genome to assist in bamboo species classification and believe that using the cp genome can provide valuable information in the classification of bamboo [14,15,16].

*D. farinosus* belongs to *Dendrocalamus*, which is mainly distributed in southwest China [17]. Compared with other species of *Dendrocalamus*, *D. farinosus* is resistant to cold and drought [18]. The bamboo shoots of *D. farinosus* have a high nutritive content, and *D. farinosus* is a high-quality material used for weaving and papermaking. With the continuous expansion of bamboo resources and the scale of development, further study of the cp genome will improve our understanding of this species, as well as assist in future breeding experiments. In this study, the complete cp genome of *D. farinosus* was assembled, and the features of this cp genome were fully elucidated. Moreover, 34 cp genomes of Bambusoideae were downloaded from GenBank and used to analyze the genealogical relationships and evolutionary history, which may allow us to better understand the identity of *D. farinosus* and the taxonomy of Bambusoideae. Moreover, this work gives us new information on the evolution and origin of Bambusoideae.

## 2. Results and Discussion

### 2.1. Characteristics of the D. farinosus cp Genome

**Figure 1:**
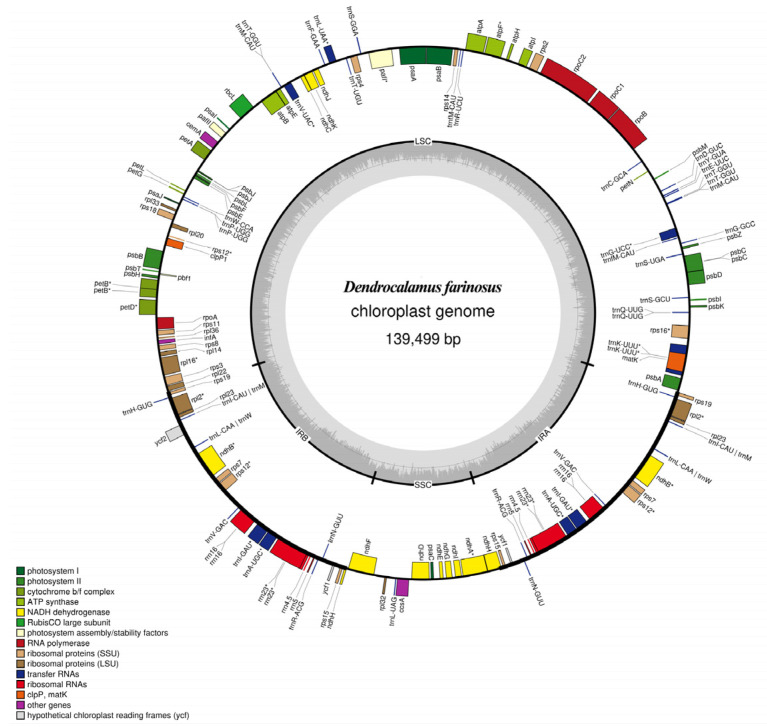
The circle gene map of the *D. farinosus* cp genome. Genes on the outside and inside the map are transcribed clockwise and counterclockwise, respectively. Genes belonging to different functional groups are color-coded. The darker grey in the inner circle corresponds to GC content. The SSC region, LSC region, and inverted repeats (IRa and IRb) are indicated.

In the current study, 3665 Mb of whole-genome raw data were developed from *D. farinosus*. After quality trimming, the data were reduced to 3128 Mb, with an average read length of 150 bp. Furthermore, we generated the complete cp genome by mapping the quality reads to the reference cp genome B. emeiensis (HQ337797), and 14.65% of the reads were mapped. The complete plastome sequence of *D. farinosus* has a circular and quadripartite structure, with a total length of 139,499 bp (GeneBankid: OM177223). The plastome has four distinct regions: a small single copy (SSC), a large single copy (LSC) and a pair of inverted repeats (IRa and IRb) (Figure 1). The gene coding region is 64,924 bp, constituting 46.54% of the genome. The length of the SSC, LSC, IRa and IRb regions were 12,879 bp (9.23%), 83,030 bp (59.52%), 21,795 bp (15.62%) and 21,795 bp (15.62%), respectively. Furthermore, the GC contents of the SSC, LSC, IRa and IRb regions were 33.2%, 37%, 44.3% and 44.3%, respectively (Table S1). The results are similar to those found in *D. sinicus*, *Ph. heteroclada*, *Ph. nidularia* and *Thyrsostachys siamensis*. The cp genomes of all five species were composed of SSC, LSC and IR regions, and the proportions of those regions in the cp genomes for all five species, were similar [19,20,21,22].

The complete cp genome of *D. farinosus* contains a total of 132 genes. Among the 132 genes are 89 protein-coding genes, eight rRNAs, and 35 tRNAs (Table 1). Furthermore, out of these 132 genes, 90 genes are present in the single-copy regions, 42 in the inverted repeat region, and the ndhH gene is present partly in both the SSC and IRa regions. In addition, the inverted repeat region contains 19 protein-coding genes, eight rRNAs and 20 tRNAs. In addition, the LSC includes 60 protein-coding genes and 23 tRNAs, while the SSC contains nine protein-coding genes and one tRNA. Hecht et al. (2017) identified that besides the canonical start codon (ATG), translation initiation with the non-canonical start codons might contribute to peptide synthesis [23]. The *D. farinosus* cp genome contains 14 genes with non-canonical start codons (Table S2). Among the 14 genes, eight genes are located in the LSC region, and six are located in the IR region. Furthermore, out of these 132 genes, 13 genes contain exons and introns. Among the 13 genes, 12 genes contain two exons and one intron. In addition, the pafI gene has three exons and two introns. Moreover, among these 13 genes, seven are distributed in the LSC, five genes occur in both the IRs and one in the SSC (Table 2). The longest intron of trnK-UUU is 2505 bp, while the shortest intron of rps12 is 537 bp. Most of the genes in the cp genome are protein-coding genes. This result is present in Bambusoideae, such as *D. sinicus*, *Ph. heteroclada*, *Ph. nidularia*, *T. siamensis*, etc., and also in other species such as *Abutilon fruticosum* and *Catha edulis* [19,20,21,22,24,25]. The trnK-UUU gene also has the longest intron in the cp genome of other species (A. fruticosum and C. edulis) [24,25].

**Figure 2:**
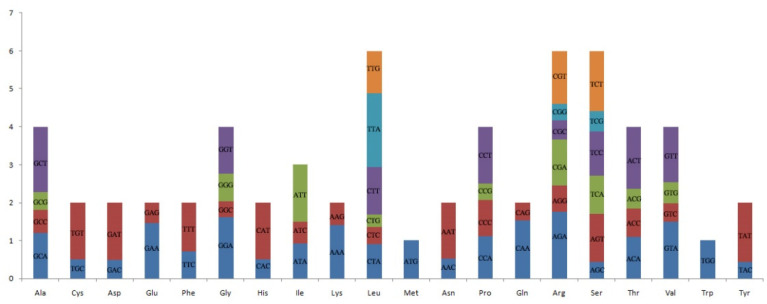
Codon content of 20 amino acids in all protein-coding genes of the *D. farinosus* cp genome.

Codon usage is believed to play a crucial role in the cp genome [26]. Codon usage is a factor shaping the evolution of cp genomes, and it varies across different species [27]. In this study, we analyzed the codon usage bias and relative synonymous codon usage (RSCU) based on the nucleotide sequence of protein-codon genes 64,924 bp, which encoded 21,023 amino acids (Table S3). The most popular type was leucine (10.85%), followed by isoleucine (8.18%) and serine (7.47%), whereas the least popular was cysteine (1.14%). The RSCU values of 30 codons were >1, and most of these codons ended with A/T (U), while for 29 codons, the values were <1, and most of them ended with G/C (Figure 2). Only two amino acids, tryptophan and methionine, showed no biased usage (RSCU = 1), which agrees with the analysis of cp genomes from other species [26,28,29].

### 2.2. Repeat Analyses

#### 2.2.1. Long Repeats

The *D. farinosus* cp genome contains three kinds of long repeats (palindromic, forward and reverse) (Table S4). The analysis showed 14 palindromic repeats, 29 forward repeats and six reverse repeats. The longest repeat was 60 bp in length, and it was a forward-type repeat located in the LSC. While the two shortest repeats, a forward and a reverse repeat, both 19 bp in length, were located in the LSC. Most of the repeats were between 20–30 bp (85.71%) in length, with 10.2% of the repeats being longer than 30 bp, and 4.08% of the repeats being shorter than 20 bp. A total of thirty-six long repeats were present in the LSC, two repeats were present in the SSC, seven long repeats were detected in two different regions, and four repeats were present in the IR regions.

#### 2.2.2. Simple Sequence Repeats (SSRs)

Simple sequence repeats (SSRs) are essential for genetic mapping, variety identification and molecular marker-assistant breeding. The *D. farinosus* cp genome contains forty-two SSRs, including seventeen mononucleotides (40.48%), four dinucleotides, three trinucleotides, nine tetranucleotides, and one pentanucleotide with a length of at least 10 bp (Table 3). In addition, the *D. farinosus* cp genome also contains eight compound SSRs (19.05%). All seventeen mononucleotides consisted of poly-A and poly-T. The AT/TA/TC types contributed to four dinucleotides, and the length of these SSRs was 10 bp. Three trinucleotide repeats were detected, namely AAT (12 bp), CAG (12 bp), and TTC (12 bp), which were located in the LSC. Moreover, the eight compound formations were also located in the LSC.

### 2.3. Comparative Analysis of Plastomes of Bambusoideae Species

**Figure 3:**
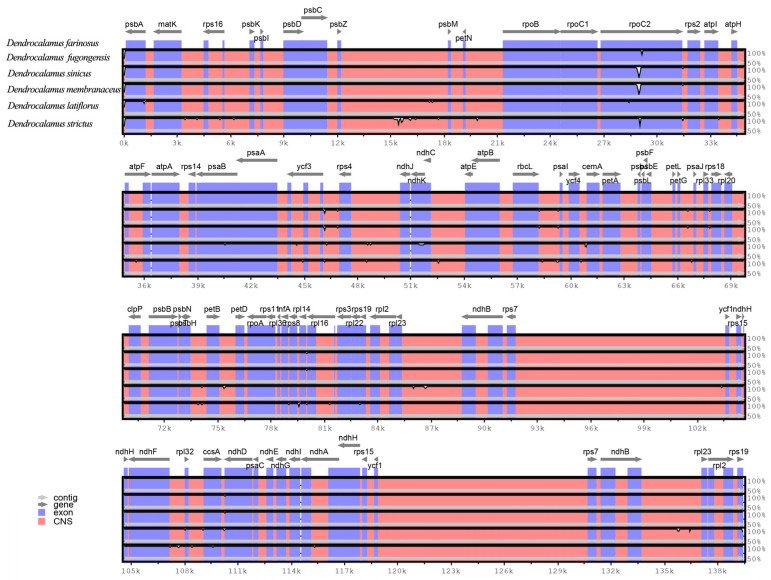
Sequence alignment of the six cp genomes of *Dendrocalamus* by mVISTA, using *D. farinosus* as the reference. The six genomes of *Dendrocalamus* are *D.* *farinosus*, *D.* *fugongensis*, *D. sinicus*, *D. membranaceus*, *D. latiflorus* and *D. strictus*. The horizontal axis indicates the coordinates within the cp genome. Genome regions are color-coded as follows; genes are represented in blue; conserved non-coding sequences are represented in dark pink; forward and backward arrows represent the direction of the gene.

**Figure 4:**
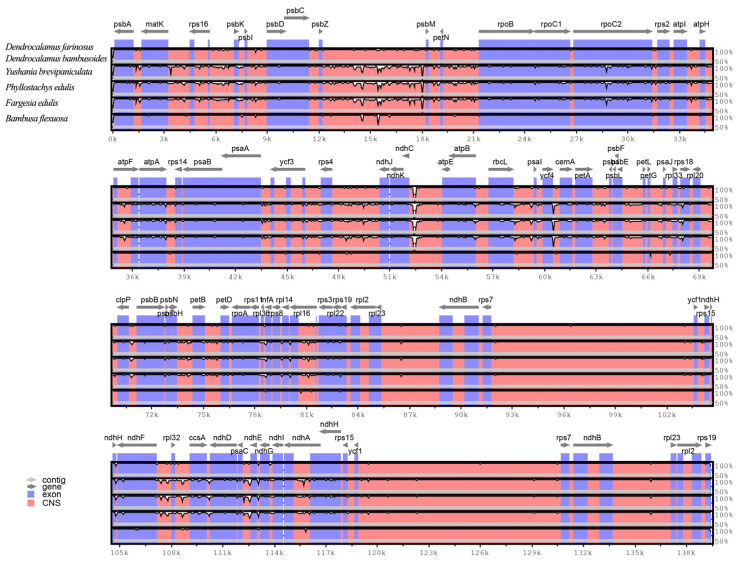
Sequence alignment of the six cp genomes of *Bambusoideae* with mVISTA using *D. farinosus* as a reference. The six genomes of *Bambusoideae* are *D. farinosus*, *D. bambusoides*, *Y. brevipaniculata*, *Ph. edulis*, *F. edulis* and *B. flexuosa*. The horizontal axis indicates the coordinates within the cp genome. Genome regions are color-coded as follows; genes are represented in blue; conserved non-coding sequences are represented in dark pink; forward and backward arrows represent the direction of the gene.

To detect divergence in the cp genome of the six species of *Dendrocalamus* (*D. farinosus*, *D. fugongensis*, *D. sinicus*, *D. membranaceus*, *D. latiflorus* and *D. strictus*), a comparative analysis was conducted by aligning the cp genome sequences using *D. farinosus* as a reference genome (Figure 3). The results showed that the cp genomes of *Dendrocalamus* were conserved primarily. The most divergent gene among the six cp genomes is *rpoC2*. Furthermore, a lower level of variability was also observed in non-coding regions. Moreover, we also compared the divergence of six cp genomes belonging to five genera in *Bambusoideae* (*D. farinosus*, *D.* *bambusoides*, *Yushania brevipaniculata*, *Ph. edulis*, *Fargesia edulis* and *B.* *flexuosa*) (Figure 4). The analysis showed that the non-coding regions were more divergent than the coding regions, and the inverted repeat regions were more conserved than the single-copy regions. Again, the most divergent gene was *rpoC2*. In addition, a lower level of variability was also observed in the following genes: *psbA, matK, psbD, rpoB, rpoC1, rps2, atpA, psaB, rps4, ndhK, atpB, rbcL, cemA, rpl22, ndhF, ccsA, ndhD* and *ndhH*. The results suggest that these genes can be used as identification loci for different genera in *Bambusoideae*, and *rpoC2* may be used to distinguish different bamboo species. Zhang et al. (2011) found that the genetic divergence is very low among the cp genomes of *Bambusoideae*, but the *rpoC2* gene is an exception [30]. This result is consistent with our study. 

**Figure 5:**
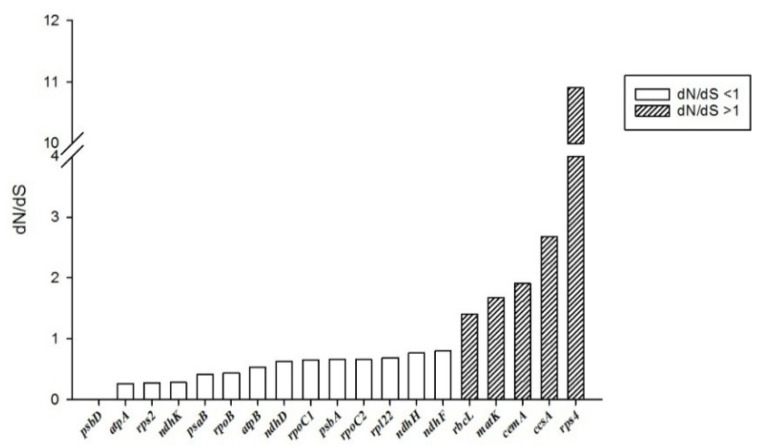
The nonsynonymous (dN)/synonymous (dS) ratio values of 19 genes from six Bambusoideae cp genomes (*D. farinosus*, *Y. brevipaniculata*, *Ph. edulis*, *F. edulis*, *D. bambusoides* and *B. flexuosa*).

The nonsynonymous (dN)/synonymous (dS) ratio was calculated for 19 genes that were observed to be divergent in the cp genomes of the Bambusoideae genera (Figure 5). The dN/dS of 13 genes (68.42%) were <1, suggesting that most of them were in negative selection. The dN/dS value of five genes, *rbcL, matK, cemA, ccsA* and *rps4*, were >1, indicating that they were in positive selection, and may provide an evolutionary advantage. Furthermore, the *psbD* gene does not contain any nonsynonymous changes in the plastome of the species used in this study.

**Figure 6:**
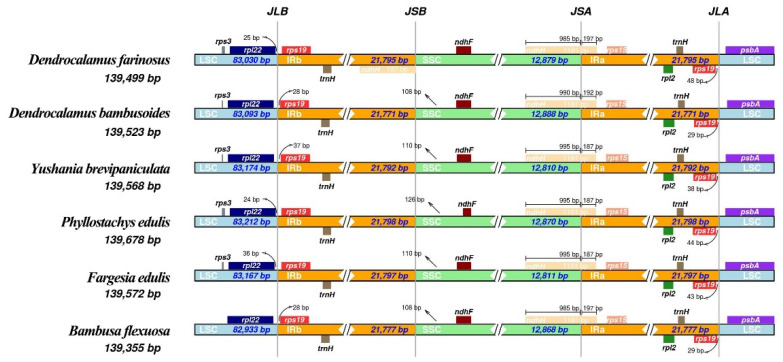
Comparison of the borders of the SSC, LSC, IRa and IRb regions among six cp genomes of Bambusoideae. JLB: junction of the LSC and the IRb. JSB: junction of the SSC and the IRb. JSA: junction of the SSC and the IRa. JLA: junction of the LSC and the IRa.

The cp genome of plants is conserved in structure and size. In contrast, the expansion and contraction in the cp genomes also occur, and the size and location of the boundaries of single-copy regions and inverted repeat regions changed slightly. This is generally considered the driving force in plant cp genome variation [26]. The boundaries of the IR-LSC and the IR-SSC of six bamboo species (five genera) were compared and analyzed (Figure 6). The size of the six cp genomes ranged from 139,355 bp (*B.* *flexuosa*) to 139,678 bp (*Ph. edulis*). The gene, ndhH, existed simultaneously at the junction of the SSC-IRa (JSA) and the junction of the SSC-IRb (JSB) in the cp genome of *D. farinosus*, while it only appeared at the JSA in the other five cp genomes, and expanded to 187–197 bp in the IRa region. The gene, *rps19*, existed at both the junction of the LSC-IRa (JLA) and the junction of the LSC-IRb (JLB), and varying degrees of contraction and expansion occurs in the cp genomes of the six species. The *rpl22* gene of *D. farinosus*, *Ph. edulis* and *F. edulis* contracted inward by 25 bp, 24 bp and 36 bp from the border, respectively. Kim et al. (2004) thought that the expansion and contraction in the cp genomes was probably mediated by the intramolecular recombination of two short direct repeat sequences which frequently occur within the genes located at the borders [31]. Therefore, the driving force for the expansion and contraction of the cp genome of *D. farinosus* may be greater than that of the other five cp genomes.

### 2.4. Phylogenetic Analysis

**Figure 7:**
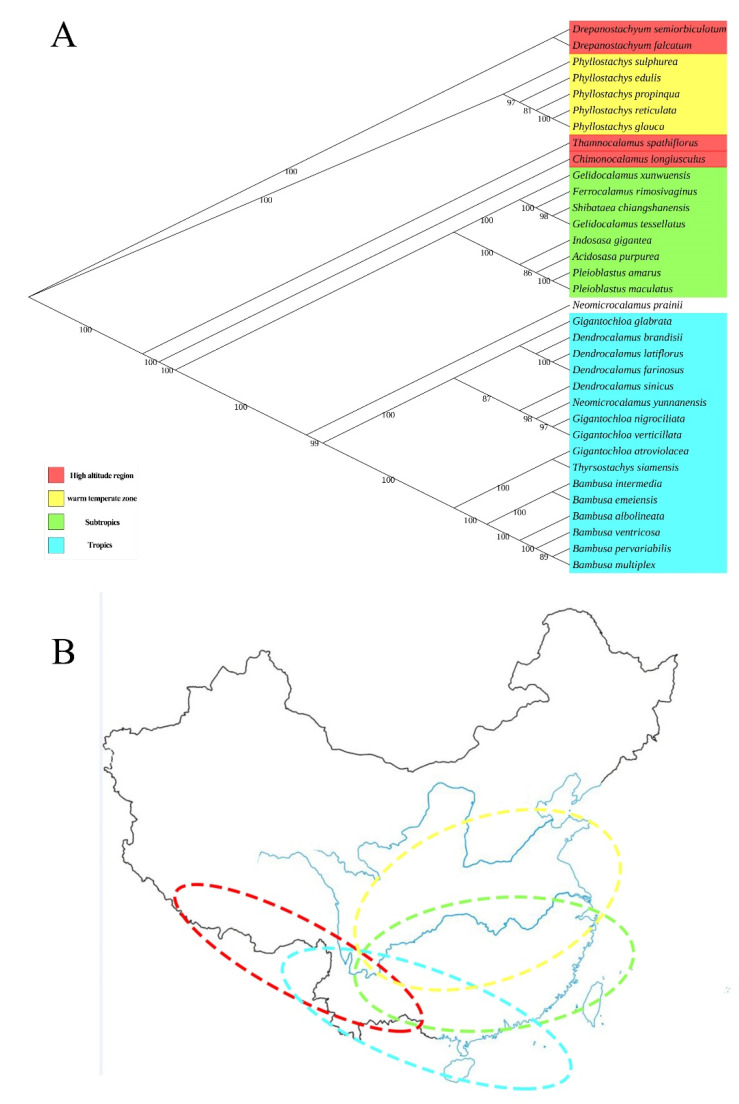
The maximum-likelihood tree based on the 19 cp genomes of Bambuseae and the distribution of the main geographical provenances in China. (**A**) The bootstrap value based on 1000 replicates is shown on each node. (**B**) Distribution of the main geographical provenances of 34 bamboo species in China. Red circle: the distribution of bamboos in high altitude regions; Yellow circle: the distribution of bamboos in the warm temperate zone; Green circle: the distribution of bamboos in the subtropics; Blue circle: the distribution of bamboos in the tropics.

The cp genomes containing a large amount of genetic information are a good resource for inferring evolutionary and phylogenetic relationships [26,32,33,34]. Many bamboo species have a similar morphology, which makes them difficult to classify. In this study, the cp genomes of 34 Bambusoideae species (15 genera) were analyzed to understand the evolutionary relationship (Figure 7A). The results showed that these 34 species were classified into four clades, and *Neomicrocalamus prainiias* was an outgroup. *D. farinosus* was divided into the same clade as other members of *Dendrocalamus*. Members of *Dendrocalamus*, *Bambusa*, and *Gigantochloa* are in the same clade along with *Neomicrocalamus yunnanensis*. These results differed from the traditional classification method, while remaining consistent with recent studies [1,35]. Members of Phyllostachys are in a clade and members of *Drepanostachyum* are in a clade. The result is consistent with the classification according to morphology, indicating that the cp genome-assisted bamboo species classification may be able to solve the complex problem of bamboo classification. As increasing numbers of cp genomes of bamboo species are sequenced, it will be more advantageous to demonstrate that this method is feasible.

The evolutionary analysis of the cp genome may provide new information about the evolution and origin of bamboos. The evolution and origin of bamboo have long been controversial. The origin of bamboo is believed to be polycentric, with two major points of origin in Asia and South America. At the same time, Wen Taihui (1983) proposed that there is only one origin center for bamboo globally, and it is located in the Yunnan province of China [36]. In this study, out of 34 bamboo species, 33 were divided into four groups: high altitude region, warm temperate zone, Subtropics and tropics. These four groups overlap at the Sichuan and Yunnan provinces. At the same time, the 34th bamboo species used in this study, *Neomicrocalamus prainii*, is mainly distributed at the junction of four regions (Sichuan province, China, located in the subtropical region, altitude of 1000–3000 m) (Figure 7B). Furthermore, all 34 bamboo species used in this study were available in the Yunnan province, which we believe may be the origin of bamboo. The results are consistent with Wen [36]. The members of *Drepanostachyum* and *Thamnocalamus* are found mainly in the vicinity of the Himalayas, where the average height above sea level is 6000 m [37]. The *Chimonocalamus* *longiusculus* is primarily found in southeastern Tibet (average altitude: 4000 m) and southwestern Yunnan (average altitude: 2000 m) [37]. The members of *Phyllostachys* are found mainly in warm temperate zones and subtropical regions [37]. Nine species are distributed in the subtropical regions, and 16 are mainly distributed in the tropics [37]. 

The comparison of the rhizomes of the 34 species revealed that all of the species of high-altitude regions and the tropics have sympodial rhizomes, and all of the species of the Subtropics involved in the comparison have amphipodial rhizomes and monopodial rhizomes. Based on the geographical distribution and rhizome types of these bamboo species, we speculate that the evolution of all the species involved in the comparison in China may be divided into tropics to alpine and tropics to warm temperate zones. The rhizomes of bamboo species that evolve to a warm temperate zone transform from sympodial to amphipodial, and then from amphipodial to monopodial. This result is consistent with the prediction of bamboo phylogeny from morphology [38]. With the completion of sequencing and further analyses of the cp genomes of more bamboo species, new evidence for these hypotheses may be provided.

## 3. Materials and Methods

### 3.1. Plant Material and DNA Extraction

Young and healthy leaves of *D. farinosus,* was collected from Changning, Sichuan province (28°29’ N, 104°58’ E). The total DNA was extracted using a modified CTAB protocol [39]. The voucher specimens are available at the College of Forestry and Biotechnology, Zhejiang A&F University (Accession No. BR01). 

### 3.2. Chloroplast Genome Assembly and Gene Annotation

The cp genome was sequenced using the Illumina NovaSeq PE150 platform (Novogene Bioinformatics Technology Co, Ltd. in Beijing, China). The complete cp genome was constructed using MITObim v1.3, and annotated using the online program, GeSeq [40]. Genes were annotated using CPGAVAS2 (Chloroplast Genome Annotation, Visualization, Analysis and GenBank Submission Tool) [41]. The circular cp genome maps were drawn using OGDRAW [42].

### 3.3. Repeat Analysis

The long repeat sequences of the *D. farinosus* cp genome were detected using REPuter [43] with default parameters. Simple sequence repeats (SSR) were detected using MISA [44] with the following settings: ten repeats for mono-types, five repeats for di-types, four repeats for tri-types, three repeats for tetra-types, three repeats for penta-types and three repeats for hexa-types.

### 3.4. Codon Bias Usage Analysis

CPGAVAS2 with default settings was used to analyze the codon bias usage of the protein-coding genes [24]. The relative synonymous codon usage (RSCU) was analyzed.

### 3.5. Comparison of Related cp Genomes

The mVISTA program was used to analyze sequence divergence between the *D. farinosus* cp genome and the cp genomes of nine related species [45]. IRscope (https://irscope.shinyapps.io/irapp/, accessed on 4 January 2022) was used to visualize the genes on the boundaries of the junction sites of the six cp genomes. MEGA 7.0 was used to estimate the dN/dS ratio to detect the genes under selection pressure [46]. 

### 3.6. Phylogenetic Analysis

The complete cp genomes of 34 Bambusoideae species were downloaded from GenBank (Table S5) and aligned using MAFFT v7 [47]. The evolutionary history was inferred using RAxML-NG with maximum likelihood, and the bootstrap consensus tree was inferred from 1000 replicates [48].

## 4. Conclusions

Our results identified the complete cp genome of *D. farinosus* and the evolutionary studies with other Bambusoideae species. Our study identified the basic structure and gene content of the cp genome of *D. farinosus*. Further, our analysis also determined that the *rpoC2* gene might be used to distinguish between different bamboo species. Moreover, these bamboo species’ geographical distribution and rhizome types indicate two evolutionary pathways: the tropics to the alpine zone and the tropics to the warm temperate zone. This study provides the cp genome data for species identification and phylogenetic analysis of the Bambusoideae family.

## Figures and Tables

**Table 1:** Genes present in the cp genome of *D. farinosus*.

Category	Group of Genes	Name of Genes
Genes for photosynthesis	Subunits of ATP synthase	*atpA, atpB, atpE, atpF* ^+^ *, atpH, atpI*
	Subunits of NADH-dehydrogenase	*ndhA* ^+^*, ndhB* ^+,a^*, ndhC, ndhD, ndhE, ndhF, ndhG, ndhH, ndhJ, ndhK*
	Subunits of cytochrome b/f complex	*petA, petB* ^+^*, petD*^+^*, petG, petL, petN*
	Subunits of photosystem I	*psaA, psaB, psaC, psaI, psaJ*
	Subunits of photosystem II	*psbB, psbC, psbD, psbE, psbF, psbH, psbI, psbJ, psbK, psbL, psbM, psbT, psbZ*
	Subunit of rubisco	*rbcL*
	photosystem assembly/stability factors	*pafI* ^++^*, pafII, pbf1*
Self replication	Large subunit of ribosome	*rpl2*^+,a^*, rpl14, rpl16, rpl20, rpl22, rpl23* ^a^*, rpl32, rpl33, rpl36*
	Small subunit of ribosome	*rps2, rps3, rps4, rps7*^a^*, rps8, rps11, rps12*^++,a^*, rps12-fragment*^+^*, rps14, rps15*^a^*, rps16* ^+^*, rps18, rps19* ^a^
	DNA dependent RNA polymerase	*rpoA, rpoB, rpoC1* ^+^ *, rpoC2*
	tRNA Genes	*trnA-UGC*^+,a^*, trnC-GCA, trnD-GUC, trnE-UUC, trnF-GAA, trnfM-CAU, trnG-GCC, trnG-UCC* ^+^*, trnH-GUG*^a^*, trnI-CAU*^a^*, trnI-GAU* ^+,a^*, trnK-UUU ^+^, trnL-CAA* ^a^*, trnL-UAA* ^+^*, trnL-UAG, trnM-CAU, trnN-GUU* ^a^*, trnP-UGG, trnQ-UUG, trnR-ACG*^a^*, trnR-UCU, trnS-GCU, trnS-GGA, trnS-UGA, trnT-GGU, trnT-UGU, trnV-GAC* ^a^*, trnV-UAC*^+^*, trnW-CCA, trnY-GUA*
	rRNA Genes	*rrn4.5*^a^*, rrn5*^a^*, rrn16* ^a^*, rrn23*^+,a^
Other genes	c-type cytochrom synthesis gene	*ccsA*
	Envelop membrane protein	*cemA*
	Protease	*clpP1*
	Translational initiation factor	*infA*
	Maturase	*matK*
	Component of TIC complex	*ycf2, ycf15* ^a^
	Subunit acetyl-coA carboxylase	*accD*

‘^+^’—Gene with one intron, ‘^++^’—gene with two introns and ‘^a^’—gene with copies.

**Table 2:** The introns in the genes of the *D. farinosus* cp genome.

S. No	Gene	Location	Exon I (bp)	Intron I (bp)	Exon II (bp)	Intron II (bp)	Exon III (bp)
1	*rps16*	LSC	36	846	222		
2	*atpF*	LSC	158	837	409		
3	*pafI*	LSC	132	732	226	724	161
4	*rpl2*	IR	405	660	432		
5	*ndhB*	IR	777	712	756		
6	*rps12*	IR	232	537	36		
7	*ndhA*	SSC	548	1026	541		
8	*trnK-UUU*	LSC	38	2505	34		
9	*trnG-UCC*	LSC	23	673	48		
10	*trnL-UAA*	LSC	35	539	50		
11	*trnV-UAC*	LSC	39	596	37		
12	*trnI-GAU*	IR	42	946	35		
13	*trnA-UGC*	IR	38	811	35		

**Table 3:** Distribution of SSRs in the *D. farinosus* cp genome.

SSR Type	Unit	Length	Number	Position on Genome (bp)
P1	A	10	4	7647–7656, 12,227–12,236, 31,304–31,313, 39,008–39,017
	A	11	3	49,902–49,912, 107,247–107,257, 139,481–139,491
	A	12	2	45,950–45,961, 46,921–46,932
	A	13	1	110,286–110,298
	T	10	4	38,212–38,221, 43,628–43,637, 67,161–67,170, 82,514–82,523
	T	11	2	34,229–34,239, 79,557–79,567
	T	12	1	51,765–51,776
P2	AT	10	1	27,587–27,596
	TA	10	2	87,344–87,353, 135,177–135,186
	TC	10	1	117,486–117,495
p3	AAT	12	1	26,292–26,303
	CAG	12	1	684–695
	TTC	12	1	67,459–67,470
P4	AAAG	12	1	18,988–18,999
	AACG	12	1	101,658–101,669
	AATA	12	1	110,503–110,514
	ATAC	12	1	17,899–17,910
	GAAA	12	1	65,129–65,140
	GTAG	16	1	53,971–53,986
	TATT	12	1	50,161–50,172
	TCCT	12	1	44,873–44,884
	TCGT	12	1	120,860–120,871
p5	TTTTA	15	1	31,515–31,529
C	(A)10/(A)11	50	1	35,536–35,585
	(T)10/(T)10	28	1	78,948–78,975
	(T)10/(T)10	32	1	8601–8632
	(T)10/(A)13	106	1	48,475–48,580
	(AGAA)3/(T)11	39	1	70,751–70,789
	(TTTA)3/(A)13	61	1	74,099–74,159
	(TCT)4/(T)11	95	1	82,955–83,049
	(T)10/(AATA)3/(TATT)3	143	1	58,342–58,484

## Data Availability

The data which supports the findings of this study are openly available in GenBank of NCBI at https://www.ncbi.nlm.nih.gov, accessed on 20 January 2022, reference number (*D. farinosus*, OM177223).

## Supplementary Materials

The following supporting information can be downloaded at: https://www.mdpi.com/article/10.3390/genes13091519/s1, Table S1: Base composition in the *D. farinosus* cp genome; Table S2: Genes with non-canonical start codons in the *D. farinosus* cp genome; Table S3: Summary of codon usage and amino acid patterns of the *D. farinosus* cp genome; Table S4: The long repeat sequences detected in the *D. farinosus* cp genome; Table S5: List of the cp genome of 34 Bambusoideae species used for phylogenetic analysis.
