# Supplementary material for: Complete Chloroplast Genome Features of Dendrocalamus farinosus and Its Comparison and Evolutionary Analysis with Other Bambusoideae Species
Source: Genes (Basel). 2022 Aug 24;13(9):1519. doi: 10.3390/genes13091519 (PMC9498922; doi:10.3390/genes13091519)
Supplement: Supplementary file 1 [file genes-13-01519-s001.zip › genes-1863871-supplementary.pdf]

Table S1. Base composition in the *D. farinosus* cp genome

Region	A (%)	T(U) (%)	C (%)	G (%)	Total length (bp)
Cp genome	30.6	30.5	19.4	19.5	139,499
LSC	31.3	31.7	18.3	18.7	83,030
SSC	35.8	31	17.1	16.1	12,879
IRa	27.9	27.9	23.1	21.2	21,795
IRb	27.9	27.9	21.2	23.1	21,795

Table S2. Genes with non-canonical start codons in *D. farinosus* cp genome

S. No	gene	initiation codon	location
1	*accD*	GGT	LSC
2	*petN*	ATT	LSC
3	*psbI*	AAA	LSC
4	*psbN*	TTG	LSC
5	*psbT*	ATC	LSC
6	*rpl16*	AAC	LSC
7	*rpl36*	TTA	LSC
8	*rps16*	GCT	LSC
9	*rpl2*	ATA	IRb
10	*rps19*	GTG	IRb
11	*rpl2*	ATA	IRa
12	*rps12*	GAG	IRa
13	*rps15*	AAA	IRa
14	*ycf15*	CGT	IRa

Table S3. Summary of codon usage and amino acid patterns of *D. farinosus* cp genome.

Codon	Number	Amino acids	Ratio of codon (%)	RSCU	Number of amino acid	Ratio of amino acid(%)
GCA	390	Ala	1.86	1.20	1,296	6.16
GCC	196		0.93	0.60		
GCG	151		0.72	0.47		
GCT	559		2.66	1.73		
TGC	61	Cys	0.29	0.51	239	1.14
TGT	178		0.85	1.49		
GAC	181	Asp	0.86	0.48	757	3.60
GAT	576		2.74	1.52		

GAA	800	Glu	3.81	1.47	1,092	5.19
GAG	292		1.39	0.53		
TTC	418	Phe	1.99	0.71	1,175	5.59
TTT	757		3.60	1.29		
GGA	622	Gly	2.96	1.61	1,543	7.34
GGC	161		0.77	0.42		
GGG	281		1.34	0.73		
GGT	479		2.28	1.24		
CAC	120	His	0.57	0.50	481	2.29
CAT	361		1.72	1.50		
ATA	523	Ile	2.49	0.91	1,719	8.18
ATC	337		1.60	0.59		
ATT	859		4.09	1.50		
AAA	769	Lys	3.66	1.41	1,090	5.18
AAG	321		1.53	0.59		
CTA	343	Leu	1.63	0.90	2,282	10.85
CTC	172		0.82	0.45		
CTG	128		0.61	0.34		
CTT	475		2.26	1.25		
TTA	740		3.52	1.95		
TTG	424		2.02	1.11		
ATG	492	Met	2.34	1.00	492	2.34
AAC	219	Asn	1.04	0.52	847	4.03
AAT	628		2.99	1.48		
CCA	252	Pro	1.20	1.11	910	4.33
CCC	217		1.03	0.95		
CCG	101		0.48	0.44		
CCT	340		1.62	1.49		
CAA	526	Gln	2.50	1.52	692	3.29
CAG	166		0.79	0.48		
AGA	386		1.84	1.76		
AGG	150		0.71	0.68		
CGA	269		1.28	1.23		
CGC	111		0.53	0.51		
CGG	93		0.44	0.42		
CGT	308		1.47	1.40		
AGC	112		0.53	0.43		
AGT	333		1.58	1.27		
TCA	266		1.27	1.02		
TCC	301		1.43	1.15		
TCG	142		0.68	0.54		
TCT	416		1.98	1.59		
ACA	316		1.50	1.10		

ACC	212		1.01	0.74		
ACG	149		0.71	0.52		
ACT	472		2.25	1.64		
GTA	460		2.19	1.50		
GTC	149		0.71	0.49		
GTG	170		0.81	0.55		
GTT	449		2.14	1.46		
TGG	372		1.77	1.00		
TAC	169		0.80	0.44		
TAT	603		2.87	1.56		

Table S4. The long repeat sequences detected in *D. farinosus* cp genome

No.	Repeat	Type	Repeat 1 Start(bp)	Repeat 2 Start(bp)	Region
1	60	F	29,090	29,135	LSC
2	58	F	28,776	28,956	LSC
3	41	F	28,847	28,931	LSC
4	31	F	14,098	84,837	LSC;IRb
5	31	P	14,098	137,661	LSC;IRa
6	30	P	18,346	18,346	LSC
7	30	F	28,739	28,760	LSC
8	30	F	68,089	68,110	LSC
9	30	P	68,468	68,468	LSC
10	30	P	89,696	89,696	IRb
11	30	F	89,696	132,803	IRb;IRa
12	30	P	132,803	132,803	IRa
13	29	P	7,903	46,752	LSC
14	29	F	28,887	28,929	LSC
15	28	F	78,658	78,676	LSC
16	27	F	12,949	13,116	LSC
17	27	F	28,847	28,889	LSC
18	26	F	28,835	29,015	LSC
19	26	F	82,243	82,288	LSC
20	25	F	28,797	29,130	LSC
21	25	F	28,977	29,130	LSC
22	25	P	104,017	104,044	IRb
23	25	F	104,017	118,460	IRb;IRa
24	25	F	104,044	118,487	IRb;IRa
25	25	P	118,460	118,487	IRa
26	24	F	15,938	16,006	LSC
27	24	P	108,424	108,454	SSC
28	22	F	14,741	92,040	LSC;IRb
29	22	P	14,741	130,467	LSC;IRa

30	21	F	7,908	11,577	LSC
31	21	P	11,577	46,755	LSC
32	21	P	15,968	47,974	LSC
33	21	R	29,120	29,120	LSC
34	21	R	29,120	29,165	LSC
35	21	R	29,165	29,165	LSC
36	20	R	6,138	6,138	LSC
37	20	F	16,074	16,094	LSC
38	20	F	26,773	26,794	LSC
39	20	F	28,797	29,175	LSC
40	20	F	28,802	29,090	LSC
41	20	F	28,977	29,175	LSC
42	20	F	28,982	29,090	LSC
43	20	F	40,389	42,613	LSC
44	20	R	51,934	51,934	LSC
45	20	F	68,058	68,079	LSC
46	20	P	72,998	73,023	LSC
47	20	P	105,348	105,348	SSC
48	19	F	8,316	8,333	LSC
49	19	R	17,896	17,896	LSC

Note: P represents for palindrome, R for reverse, F for forward, and C for complement types

Table S5. List of the cp genome of 34 Bambusoideae species used for phylogenetic analysis.

Latin Name	Genus	NCBI Accession
Acidosasa purpurea	*Acidosasa*	HQ337793
Bambusa albolineata	*Bambusa*	MW557324
Bambusa emeiensis	*Bambusa*	HQ337797
Bambusa intermedia	*Bambusa*	MW463057
Bambusa multiplex	*Bambusa*	NC_024668
Bambusa pervariabilis	*Bambusa*	NC_053748
Bambusa ventricosa	*Bambusa*	NC_042671
Chimonocalamus longiusculus	*Chimonocalamus*	NC_024714
Dendrocalamus brandisii	*Dendrocalamus*	NC_050763
Dendrocalamus farinosus	*Dendrocalamus*	OM177223
Dendrocalamus latiflorus	*Dendrocalamus*	FJ970916
Dendrocalamus sinicus	*Dendrocalamus*	NC_045941
Drepanostachyum falcatum	*Drepanostachyum*	NC_037167
Drepanostachyum semiorbiculatum	*Drepanostachyum*	MN310559
Ferrocalamus rimosivaginus	*Ferrocalamus*	HQ337794
Gelidocalamus tessellatus	*Gelidocalamus*	NC_024719
Gelidocalamus xunwuensis	*Gelidocalamus*	MN205550
Gigantochloa atroviolacea	*Gigantochloa*	NC_050777
Gigantochloa glabrata	*Gigantochloa*	MK679788

Gigantochloa nigrociliata	*Gigantochloa*	NC_050778
Gigantochloa verticillata	*Gigantochloa*	MN688203
Indosasa gigantea	*Indosasa*	NC_046587
Neomicrocalamus prainii	*Neomicrocalamus*	NC_050769
Neomicrocalamus yunnanensis	*Neomicrocalamus*	NC_050767
Phyllostachys edulis	*Phyllostachys*	MW007170
Phyllostachys glauca	*Phyllostachys*	NC_051535
Phyllostachys propinqua	*Phyllostachys*	JN415113
Phyllostachys reticulata	*Phyllostachys*	MN537808
Phyllostachys sulphurea	*Phyllostachys*	KJ722540
Pleioblastus amarus	*Pleioblastus*	NC_043892
Pleioblastus maculatus	*Pleioblastus*	NC_024723
Shibataea chiangshanensis	*Shibataea*	NC_036826
Thamnocalamus spathiflorus	*Thamnocalamus*	NC_024724
Thyrsostachys siamensis	*Thyrsostachys*	NC_024724
